# Clinical performance of an antibody-free assay for plasma Aβ42/Aβ40 to detect early alterations of Alzheimer’s disease in individuals with subjective cognitive decline

**DOI:** 10.1186/s13195-022-01143-z

**Published:** 2023-01-05

**Authors:** María Pascual-Lucas, José Antonio Allué, Leticia Sarasa, Noelia Fandos, Sergio Castillo, Jose Terencio, Manuel Sarasa, Juan Pablo Tartari, Ángela Sanabria, Lluís Tárraga, Agustín Ruíz, Marta Marquié, Sang Won Seo, Hyemin Jang, Mercè Boada, on behalf of the FACEHBI study group, N. Aguilera, E. Alarcón-Martín, M. Alegret, S. Alonso-Lana, M. Berthier, U. Bojayrin, M. Buendia, S. Bullich, F. Campos, A. Cano, P. Cañabate, L. Cañada, C. Cuevas, I. de Rojas, S. Diego, A. Espinosa, E. Esteban-De Antonio, A. Gailhajenet, A García-Sánchez, P. García, J. Giménez, M. Gómez-Chiari, M. Guitart, I. Hernández, M. Ibarria, A. Lafuente, N. Lleonart, F. Lomeña, E. Martín, M. Moreno, A. Morera, L. Montrreal, N. Muñoz, L. Narvaiza, A. Niñerola, A. B. Nogales, L. Núñez, A. Orellana, G. Ortega, A. Páez, A. Pancho, E. Pelejà, E. Pérez, A. Pérez-Cordon, A. Perissinotti, S. Preckler, V. Pytel, M. Ricciardi, O. Rodríguez-Gomez, N. Roé-Vellvé, M. I. Ramis, M. Rosende-Roca, S. Seguer, O. Sotolongo-Grau, A. Stephens, M. A. Tejero, M. Torres, S. Valero, L. Vargas, A. Vivas

**Affiliations:** 1 Araclon Biotech-Grifols, Zaragoza, Spain; 2 Ace Alzheimer Center Barcelona - Universitat Internacional de Catalunya, Barcelona, Spain; 3 CIBERNED, Network Center for Biomedical Research in Neurodegenerative Diseases, National Institute of Health Carlos III, Madrid, Spain; 4 Department of Neurology, Samsung Medical Center, Sungkyunkwan University School of Medicine, Seoul, South Korea

**Keywords:** Alzheimer’s disease, Amyloid, Aβ42/Aβ40, Ratio, Biomarkers, Plasma, Blood biomarkers, Mass spectrometry, Subjective cognitive decline

## Abstract

**Background:**

Accessible and cost-effective diagnostic tools are urgently needed to accurately quantify blood biomarkers to support early diagnosis of Alzheimer’s disease (AD). In this study, we investigated the ability of plasma amyloid-beta (Aβ)42/Aβ40 ratio measured by an antibody-free mass-spectrometric (MS) method, ABtest-MS, to detect early pathological changes of AD.

**Methods:**

This cohort study included data from the baseline and 2-year follow-up visits from the Fundació ACE Healthy Brain Initiative (FACEHBI) study. Plasma Aβ42/Aβ40 was measured with ABtest-MS and compared to ^18^F-Florbetaben PET as the reference standard (cutoff for early amyloid deposition of 13.5 centiloids). Cross-validation was performed in an independent DPUK-Korean cohort. Additionally, associations of plasma Aβ42/Aβ40 with episodic memory performance and brain atrophy were assessed.

**Results:**

The FACEHBI cohort at baseline included 200 healthy individuals with subjective cognitive decline (SCD), of which 36 (18%) were Aβ-PET positive. Plasma Aβ42/Aβ40 levels were significantly lower in Aβ-PET positive individuals (median [interquartile range, IQR], 0.215 [0.203–0.236]) versus Aβ-PET negative subjects (median [IQR], 0.261 [0.244–0.279]) (*P* < .001). Plasma Aβ42/Aβ40 was significantly correlated with Aβ-PET levels (rho = −0.390; *P* < .001) and identified Aβ-PET status with an area under the receiver operating characteristic curve (AUC) of 0.87 (95% confidence interval [CI], 0.80–0.93). A cutoff for the Aβ42/Aβ40 ratio of 0.241 (maximum Youden index) yielded a sensitivity of 86.1% and a specificity of 80.5%. These findings were cross-validated in an independent DPUK-Korean cohort (AUC 0.86 [95% CI 0.77–0.95]). Lower plasma Aβ42/Aβ40 ratio was associated with worse episodic memory performance and increased brain atrophy. Plasma Aβ42/Aβ40 at baseline predicted clinical conversion to mild cognitive impairment and longitudinal changes in amyloid deposition and brain atrophy at 2-year follow-up.

**Conclusions:**

This study suggests that plasma Aβ42/Aβ40, as determined by this MS-based assay, has potential value as an accurate and cost-effective tool to identify individuals in the earliest stages of AD, supporting its implementation in clinical trials, preventative strategies and clinical practice.

**Supplementary Information:**

The online version contains supplementary material available at 10.1186/s13195-022-01143-z.

## Background

Alzheimer’s disease (AD) is the most common form of dementia affecting 55 million people worldwide in 2021 [1]. The manifestation of clinical symptoms in AD is preceded by a long preclinical phase where cognitively normal individuals present neuropathological changes in the brain. In this context, supporting biomarker information is particularly important to assist diagnosis and prognosis of at-risk individuals.

The earliest pathological hallmark of AD, brain amyloid-β (Aβ) deposition, can be reliably identified by two well-established methods: cerebrospinal fluid (CSF) and positron emission tomography (PET)-based Aβ measures [2, 3]. However, the widespread implementation of these biomarkers to facilitate patient screening in clinical trials or in routine clinical practice is hampered by their invasiveness, costs and limited availability. Thus, more accessible and cost-effective diagnostic approaches, such as blood-based biomarkers, are urgently needed.

The reliable measurement of Aβ in plasma results technically challenging due to the low abundance of the peptides in a complex matrix such as plasma [4]; therefore, highly sensitive, accurate and robust assays are desirable. In recent years, technological advances have made possible the accurate and robust quantification of plasma Aβ40 and Aβ42. Multiple assays, either immunoassays or mass-spectrometry (MS)-based methods, have proved that plasma Aβ42/Aβ40 ratio is an accurate surrogate biomarker of brain amyloid pathology [5–8]. However, recent round robin studies have found discrepancies in the quantification of plasma Aβ40 and specially Aβ42 among different assays [9]. In addition, emerging evidence has suggested that MS-based methods identify brain Aβ deposition more accurately than immunoassays [10–12], probably because they are less susceptible to the matrix effect present in such a complex fluid. Nevertheless, immunoprecipitation-MS (IP-MS) methods are elaborate, high-cost and present modest throughput, which limit their accessibility in clinical trials or routine clinical practice.

To overcome these challenges, Araclon Biotech has developed a novel antibody-free HPLC-MS/MS method (ABtest-MS) for the quantification of plasma Aβ42/Aβ40 by performing a direct extraction of Aβ peptides from plasma. This innovative procedure requires neither IP nor digestion steps, which significantly reduces time and costs, and ultimately results in a more affordable and accessible assay. The main differential characteristics of ABtest-MS with respect to other MS-based assays are summarized in Supplementary Table 1.

In this study, we aimed to validate the clinical utility of plasma Aβ42/Aβ40, as determined with ABtest-MS, by evaluating its predictive ability to detect brain amyloid deposition in healthy individuals with subjective cognitive decline (SCD) from the Fundació ACE Healthy Brain Initiative (FACEHBI) cohort [13]. These findings have been cross-validated in an independent DPUK-Korean cohort. Furthermore, we have tested the association of ABtest-MS measures of plasma Aβ42/Aβ40 with cognitive performance and brain atrophy. Finally, we have explored the ability of plasma Aβ42/Aβ40 to predict clinical progression to mild cognitive impairment (MCI) and longitudinal changes in amyloid deposition and brain atrophy at 2 years of follow-up.

## Methods

### Study participants

The study included 200 individuals from the FACEHBI cohort, a convenience sample which comprises subjects diagnosed with SCD embedded in a long-term single-centre prospective observational study of cognition, biomarkers and lifestyle, performed at Ace Alzheimer Center Barcelona. SCD was defined as the coexistence of cognitive complaints (a score of ≥ 8 on the Spanish Modified Questionnaire of Memory Failures Every day [MFE-30]) with normal performance on a comprehensive neuropsychological battery. Further information on study design and specific inclusion/exclusion criteria have been described in detail elsewhere [13]. All subjects underwent a complete neurological and neuropsychological examination, a set of self-administered questionnaires and a battery of multimodal biomarkers, including apolipoprotein E (*APOE*) genotyping, magnetic resonance imaging (MRI) and ^18^F-Florbetaben-PET (FBB-PET). Detailed description of these procedures is provided below and in the Supplementary Information. For the present study, data from baseline visit and 2-year follow-up (V2) were analysed.

### Blood sampling and plasma Aβ measurements

Blood samples were collected in polypropylene vials with ethylenediaminetetraacetic acid (EDTA) and immediately refrigerated. Samples were 30 min centrifuged (4000 g) within 24 h from extraction to collect the plasma and then aliquoted and frozen at −80 °C until analysis according to recommended plasma handling standardized operating procedures [14].

Full-length Aβ1-40 and Aβ1-42 concentrations were measured from 200 μl of plasma using ABtest-MS, a novel antibody-free liquid chromatography-differential mobility spectrometry-triple quadrupole mass spectrometric (HPLC-DMS-MS/MS) method (Araclon Biotech, Zaragoza, Spain). Calibration curves were prepared in human plasma after spiking ^15^N-Aβ40 and ^15^N-Aβ42 (rPeptide, Watkinsville, GA, USA) at seven concentration levels. Calibration ranges were 50–1000 pg/ml for ^15^N-Aβ40 and 10-200 pg/ml for ^15^N-Aβ42. Two identical calibration curves were used in each analytical run, one at the beginning and one at the end of the sequence, in order to correct for potential analytical drift during the run time. Additionally, three quality control samples (per duplicate) were also prepared by spiking three concentration levels (high: 750/150, mid: 400/75 and low: 150/30 pg/ml for ^15^N-Aβ40 and ^15^N-Aβ42, respectively) in human plasma. Quality control samples were uniformly distributed along the sequence in each run. Analytes were extracted directly from plasma as no IP procedure was followed. Intact Aβ40 and Aβ42 species were measured as no enzymatic digestion was performed. 10 μl of deuterated internal standard solution (^2^H-Aβ40 and ^2^H-Aβ42, Bachem AG, Bubendorf, Switzerland) were spiked in all samples and response ratios corresponding to endogenous species in study samples (^14^N-Aβ40/^2^H-Aβ40 and ^14^N-Aβ42/^2^H-Aβ42) were interpolated in the mean calibration curve of each analytical run.

The analytical platform was composed of a QTRAP 6500^+^ hybrid linear ion trap-triple quadrupole mass spectrometer, fitted with a differential mobility spectrometry interface (DMS, SelexION^+^) and an IonDrive Turbo V Ions Source, coupled to a M3 Micro LC system (all from Sciex, Framingham, MA, USA). Analyst 1.6.3 software (Sciex) was used for data acquisition, while MultiQuant 3.0.3 software (Sciex) was used for data processing.

Extracts were loaded on a YMC Triart C18, 0.3 × 5mm, 5 μm trap column (YMC, Kyoto, Japan) at 50 μl/min for 1 min in 5% acetonitrile (AcN) 0.1% formic acid (FA). After the loading step, analytes were eluted and separated on a HALO ES-C18, 400Å, 3.4 μm, 0.3× 50 mm analytical column (Advanced Materials Technology, Wilmington, DE, USA) kept at 50°C in a column oven. Mobile phase A was 0.1% FA in water and mobile phase B was 0.1% FA in AcN. A linear gradient from 15% to 40% B in 3 min was used for separation. Total gradient time was 5.5 min, and total cycle time was 9 min (trapping + separation + column regeneration). The mass spectrometer was operated in positive ion mode and multiple reaction monitoring (MRM) acquisition mode. MRM transitions for ^15^N, ^14^N (endogenous) and ^2^H-Aβ species were monitored. Dwell time was set at 45 ms. MS acquisition time was 5 min after sample elution from the trap column. Suitability test samples were analysed every day at the beginning of the analytical run in order to check system performance and equal transmission for light (^14^N) and heavy (^15^N) species.

Data of sensitivity and parallelism of ABtest-MS are provided in Supplementary Table 2.

All liquid chromatography-mass spectrometry analyses were performed by personnel who were blind to participant information.

### MRI acquisition

MRI scans were performed on a 1.5-T Siemens^©^ Magneton Aera (Erlangen, Germany) using a 32-channel head coil. Anatomical T1-weighted images were acquired using a rapid acquisition gradient-echo 3D magnetization-prepared rapid gradient-echo (MPRAGE) sequence with the following parameters: repetition time (TR) 2.200 ms, echo time (TE) 2.66 ms, inversion time (TI) 900 ms, slip angle 8°, field of view (FOV) 250 mm, slice thickness 1 mm, and isotropic voxel size 1 × 1 × 1 mm. Subjects also received axial T2-weighted, 3D isotropic fast fluid-attenuated inversion recovery (FLAIR) and axial T2*-weighted sequences to detect significant vascular pathology or microbleeds. Brain atrophy was assessed using ventricular and hippocampal volume data normalized by total intracranial volume.

### FBB-PET acquisition

FBB-PET scans were obtained with a Siemens^©^ Biograph molecular-CT machine. PET images were acquired in 20 min starting from 90 min after intravenous administration of 300 Mbq of 18F-Florbetaben radio tracer (NeuraCeq^©^), administered as a single slow intravenous bolus (6 s/ml) in a total volume of up to 10 ml. MRI cortical and subcortical segmentation of the T1-weighted images was carried out with Freesurfer 5.3 (https://surfer.nmr.mgh.harvard.edu/). FBB-PET scans were processed with the FSL 5.0 package (https://fsl.fmrib.ox.ac.uk/fsl/fslwiki). FBB-PET images were coregistered onto structural images, and the standard uptake value ratio (SUVR) was determined as the mean value of the cortical regions segmented on MRI, and normalized by the cerebellum as the reference region. Centiloid (CL) values were calculated as previously described [15]. Cutoff for FBB-PET positivity was established at > 13.5 CL corresponding to early amyloid deposition [16]. All images underwent an automated de-identification process prior to analysis.

### Independent validation cohort

The DPUK-Korean cohort was enrolled in the memory clinic at Samsung Medical Center (Seoul, Korea) between 2017 and 2019. Participants classified as old controls (OC, cognitively unimpaired [CU] individuals older than 45 years) were included in the validation cohort. OCs were defined to have normal cognition on neuropsychological tests [17] without a history of neurological or psychiatric disorders. Specific inclusion/exclusion criteria, Aβ-PET imaging acquisition and analysis, and plasma procedures are described elsewhere [18]. This study was approved by the institutional review board of Samsung Medical Center.

### Statistical analysis

All statistical analyses and data visualization were carried out using GraphPad Prism v5.03 (GraphPad Software, San Diego, CA, USA), SPSS v18 (IBM, Armonk, NY, USA) or MedCalc v20.015 (MedCalc Software, Ostend, Belgium). Group differences were examined using the Mann-Whitney and Chi-square tests for continuous and categorical variables, respectively. Multiple comparisons were assessed with the Kruskal-Wallis test followed by the Dunn’s pairwise test with adjustment for multiple comparisons (Bonferroni correction). Spearman correlation coefficient (rho) was employed to investigate correlations between continuous variables. Logistic regression models and receiver operating characteristics (ROC) curves were constructed to evaluate the discriminative accuracy of brain amyloid deposition. Aβ-PET imaging was chosen as the reference standard for comparative purposes as it is a recognized biomarker of brain amyloid burden. Area under the ROC curve (AUC) of different models were compared using DeLong test. External validation across independent cohorts was performed by testing the model (estimates and intercept) derived in FACEHBI, on the DPUK-Korean cohort. To determine the association between plasma Aβ42/Aβ40 and longitudinal measures of cognition, brain amyloid deposition and brain atrophy, participants were classified as plasma Aβ42/Aβ40(+) or Aβ42/Aβ40(−) by applying a cutoff of 0.241 corresponding to the maximum Youden index. Kaplan-Meier analysis with log-rank test was performed to determine the progression to MCI or Aβ-PET(+) status at 2-year follow-up. Two-sided *P* < .05 was considered statistically significant.

## Results

### Participant characteristics

Demographic and clinical characteristics of study participants from the FACEHBI cohort at baseline are presented in Table 1. In total, 200 participants were included in the study, of which 36 (18%) were classified as Aβ-PET(+) according to the cutoff for early Aβ-PET positivity [16]. The median interval between plasma collection and Aβ-PET scans was 22 days (interquartile range [IQR] 15–43 days). The median age of the population was 67.0 years (IQR 60.0–70.0), being Aβ-PET(+) individuals older than Aβ-PET(−) subjects (*P* < .001). Sex and *APOE* ε4 number of alleles were differentially distributed between Aβ-PET(+) and Aβ-PET(−) groups (*P* = .02 and *P* < .001, respectively). As previously described [19], the performance of Aβ-PET(+) participants on the Spanish version of the Face-Name Associative Memory Exam (S-FNAME) and the derived composite S-FNAME Name (SFN-N) was significantly worse than that of Aβ-PET(−) subjects (*P* = .001 and *P* < .001, respectively).

**Table 1:** Participant characteristics of the FACEHBI cohort at baseline^a^

Characteristic	Aβ-PET(−)^b^	Aβ-PET(+)^b^	***P*** value
**Participants**, No. (%)	164 (82)	36 (18)	
**Age**, years	66.0 (60.0-69.5)	70.0 (67.0–73.0)	**.0003**
**Female**, No. (%)	110 (67)	16 (44)	**.0185**
***APOE*** **ɛ4**, No. (%)			
0 alleles	131 (80)	17 (47)	**.0002**
1 allele	29 (18)	18 (50)	
2 alleles	4 (2)	1 (3)	
**Duration of education**, years	15.0 (12.0–18.0)	16.0 (10.0–18.0)	.9796
**FBB-PET**, CL	−3.7 (−7.9–1.7)	34.1 (20.3–57.0)	**< .0001**
**MMSE**, score	29 (29–30)	30 (29–30)	.1321
**S-FNAME**, score	33.5 (22.0–46.0)	24.0 (16.0–33.0)	**.0013**
**SFN-N composite**, score	−0.03 (−0.61–0.65)	−0.88 (−1.05 to −0.22)	**< .0001**
**Ventricular volume**^c,d^,** mm**^3^	24959.5 (19776.4–32028.2)	29061.4 (21966.7–38034.6)	.1079
**Hippocampal volume**^c,d^,** mm**^3^	3606.1 (3421.0–3820.8)	3581.8 (3284.2–3792.7)	.3611
**Plasma Aβ40**^e^, pg/ml	269.3 (243.9–294.2)	297.0 (267.2–317.6)	**.0009**
**Plasma Aβ42**^e^, pg/ml	70.2 (63.1–77.3)	63.4 (57.8–72.1)	**.0041**
**Plasma Aβ42/Aβ40,** ratio	0.261 (0.244–0.279)	0.215 (0.203–0.236)	**< .0001**

*Abbreviations: APOE* apolipoprotein E, *CL* centiloid, *FBB-PET* ^18^F-Florbetaben-PET, *MMSE* Mini-Mental State Examination, *S-FNAME* Spanish version of the Face-Name Associative Memory Exam, *SFN-N* S-FNAME-Name

*P* values in bold correspond to statistically significant results

^a^ Data are median values (interquartile range), except for the variables participants, female and *APOE* ɛ4 number of alleles which are the number of cases (%). Differences between Aβ-PET(−) and Aβ-PET(+) groups were tested using Mann-Whitney and Chi-square tests, as appropriate

^b^ Aβ-PET status was defined using the cutoff established at 13.5 CL corresponding to early amyloid deposition [16]

^c^ *N*=198, data from two participants were lost due to MRI acquisition problems

^d^ Data correspond to regional volume corrected by total intracranial volume

^e^ Full-length intact Aβ1-40 and Aβ1-42 were quantified by ABtest-MS

### Association of plasma Aβ42/Aβ40 with early brain amyloid deposition

**Fig. 1:**
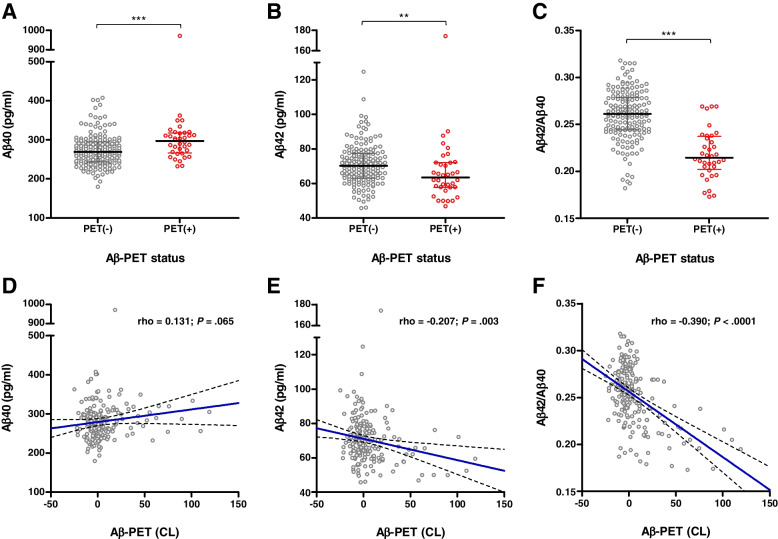
Association of plasma Aβ levels with brain amyloid deposition. **A**–**C** Distribution of plasma Aβ40 (**A**), Aβ42 (**B**) and Aβ42/Aβ40 (**C**) levels between Aβ-PET(−) and Aβ-PET(+) groups. Plasma Aβ levels were compared between Aβ-PET(−) and Aβ-PET(+) groups using Mann-Whitney test. ** *P* < .01; *** *P* < .001. Horizontal line depicts median and whiskers depict interquartile range. **D**–**F** Correlations between Aβ-PET CL and Aβ40 (**D**), Aβ42 (**E**) and Aβ42/Aβ40 (**F**) levels. Solid blue line represents the regression line; dashed lines represent 95% confidence interval. Abbreviations: CL, centiloid

Aβ40 and Aβ42 plasma levels were quantified by ABtest-MS with high accuracy and precision according to analytical performance results (Supplementary Tables 3 and 4). Significant differences were found for Aβ40, Aβ42 and Aβ42/Aβ40 plasma levels between the Aβ-PET(+) and Aβ-PET(−) groups (*P* < .001 for Aβ40 and Aβ42/Aβ40, *P* = .004 for Aβ42) (Table 1, Fig. 1A–C). Aβ42 and Aβ42/Aβ40 plasma levels showed significant negative correlations with Aβ-PET CL values (rho = −0.207, *P* = .003 for Aβ42 and rho = −0.390, *P* < .001 for Aβ42/Aβ40) (Fig. 1E, F).

### Plasma Aβ42/Aβ40 discriminative ability of Aβ-PET status

**Fig. 2:**
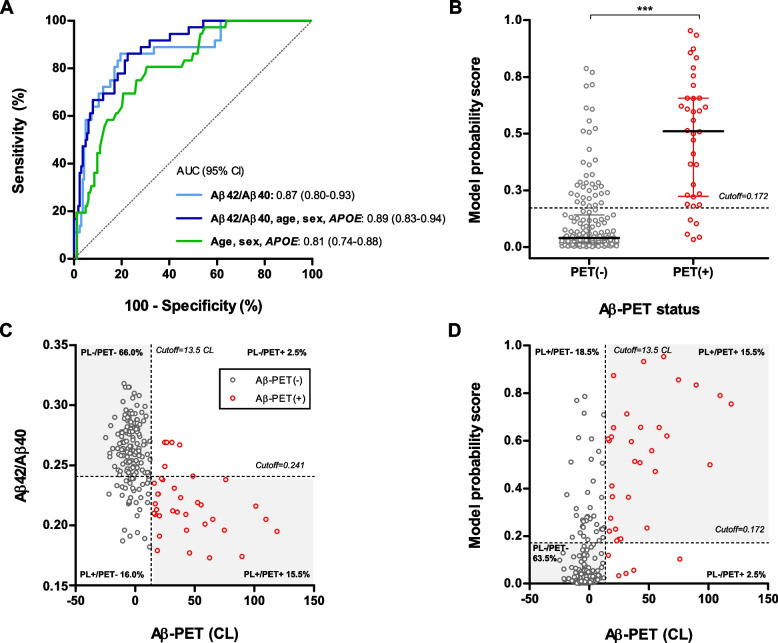
Plasma Aβ42/Aβ40 discriminative ability of Aβ-PET status. **A** Receiver operating characteristic (ROC) curves for discriminating Aβ-PET status. ROC curves are shown for plasma Aβ42/Aβ40, plasma Aβ42/Aβ40 adjusted with age, sex and *APOE* ɛ4 number of alleles, and the demographic model including only age, sex and *APOE* ɛ4 number of alleles. **B** Probability scores distribution derived from the full logistic regression model (Aβ42/Aβ40, age, sex, *APOE* ɛ4 number of alleles) to predict Aβ-PET status, between Aβ-PET(−) and Aβ-PET(+) groups. Model probability scores were compared between Aβ-PET(−) and Aβ-PET(+) groups using Mann-Whitney test. *** *P* < .001. Horizontal line depicts median and whiskers depict interquartile range. **C**, **D** Concordance plots for plasma Aβ42/Aβ40 levels (**C**) or full model probability scores (**D**) and Aβ-PET CL. Black dots correspond to Aβ-PET(−) individuals; red dots correspond to Aβ-PET(+) individuals. Dashed vertical lines represent the CL cutoff for Aβ-PET positivity. Dashed horizontal lines represent the cutoffs for plasma Aβ42/Aβ40 or the model probability score based on maximum Youden index derived by ROC analyses. Concordant classification is represented by the grey area. Abbreviations: *APOE*, apolipoprotein E; AUC, area under the curve; CI, confidence interval; CL, centiloid; PL, plasma

Plasma Aβ42/Aβ40 ratio predicted Aβ-PET status accurately with an AUC of 0.87 (95% confidence interval [CI] 0.80–0.93) (Fig. 2A). A cutoff for the Aβ42/Aβ40 ratio of 0.241, corresponding to the maximum Youden index, yielded a sensitivity of 86.1% and a specificity of 80.5% (Table 2). Concordance rate was 81.5% and, notably, among discordant cases, 32/37 (86.5%) were plasma(+)/PET(−) (Fig. 2C).

**Table 2:** Performance of logistic regression models in predicting Aβ-PET status

Model	AUC	95% CI	Se	Sp	Acc	PPV	NPV	***P*** value^a^
**Aβ42/Aβ40**	0.87	0.80–0.93	86.1%	80.5%	81.5%	49.2%	96.4%	.1544
**Aβ42/Aβ40, age, sex,** ***APOE***	0.89	0.83–0.94	86.1%	77.4%	79.0%	45.6%	96.2%	**.0054**
**Age, sex,** ***APOE***	0.81	0.74–0.88	80.6%	69.5%	71.5%	36.7%	94.2%	-

*Abbreviations: Acc* accuracy, *APOE* apolipoprotein E, *AUC* Area under the ROC curve, *CI* confidence interval, *NPV* negative predictive value, *PPV* positive predictive value, *Se* sensitivity, *Sp* specificity

*P* values in bold correspond to statistically significant results

^a^ *P* values correspond to the comparison of the AUCs of the Aβ42/Aβ40 model and the full model (Aβ42/Aβ40, age, sex, *APOE* ɛ4 number of alleles) with the AUC of the model comprising age, sex and *APOE* ɛ4 number of alleles, using the DeLong test

ROC curve analyses of plasma Aβ40 and Aβ42 showed modest discriminating performance (Supplementary Fig. 1) and therefore, only Aβ42/Aβ40 ratio was used for subsequent analyses.

Accuracy of the plasma Aβ42/Aβ40 ratio for predicting Aβ-PET status was further examined by adding age, sex and *APOE* ε4 number of alleles as covariates in the logistic regression model (Fig. 2A, B and D). The median probability score of the full model was higher among Aβ-PET(+) than Aβ-PET(−) participants (*P* < .001) (Fig. 2B). The AUC of the adjusted model increased to 0.89 (95% CI 0.83–0.94) and significantly outperformed the base model including only demographic covariates (ΔAUC = 0.08, *P* = .005) (Table 2). Both age (*P* = .012) and *APOE* ε4 number of alleles (*P* = .033) were significant predictors in this model.

Clinical performance of ABtest-MS was confirmed by ROC analysis of the 2-year follow-up visit (Supplementary Table 5). Plasma Aβ42/Aβ40 ratio identified Aβ-PET status with an AUC of 0.86 (95% CI 0.80–0.93). After adjusting with covariates, AUC was further significantly increased to 0.90 (95% CI 0.85–0.96) (ΔAUC = 0.04, *P* = .047) (Supplementary Fig. 2).

### Cross-validation in an independent cohort

To assess the generalizability of the FACEHBI results, we performed an external cross-validation of the predictive model in an independent and trans-ethnic cohort from the DPUK-Korean study. The validation cohort consisted of 148 CU individuals, of which 17 (11%) were classified as Aβ-PET(+) according to the previously published cutoff of 25.11 direct comparison centiloid units (dcCL) [18]. Participant characteristics of the DPUK-Korean cohort are shown in Supplementary Table 6.

**Fig. 3:**
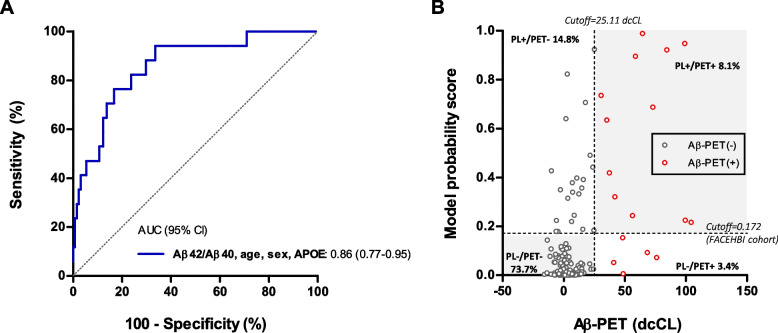
Cross-validation in an independent cohort. **A** Receiver operating characteristic (ROC) curve for discriminating Aβ-PET status in the DPUK-Korean cohort after applying the estimates and intercept of the model established in FACEHBI that included plasma Aβ42/Aβ40, age, sex and *APOE* ɛ4 number of alleles. **B** Concordance plot for the model probability scores of the validation model and Aβ-PET dcCL. Black dots correspond to Aβ-PET(−) individuals; red dots correspond to Aβ-PET(+) individuals. Dashed vertical line represents the dcCL cutoff for Aβ-PET positivity of the DPUK-Korean cohort. Dashed horizontal line represents the cutoff for the model probability score based on maximum Youden index derived by ROC analyses in FACEHBI. Concordant classification is represented by the grey area. Abbreviations: *APOE*, apolipoprotein E; AUC, area under the curve; CI, confidence interval; dcCL: direct comparison centiloid units; PL, plasma

When applying the estimates and intercept established in FACEHBI, we found that plasma Aβ42/Aβ40 adjusted with covariates (age, sex and *APOE* ε4 number of alleles) discriminated Aβ-PET status in the validation cohort with an AUC of 0.86 (95% CI 0.77–0.95) and an overall accuracy of 81.8% (Fig. 3).

### Association of plasma Aβ42/Aβ40 with episodic memory performance and brain atrophy

Participants from the FACEHBI cohort were classified as plasma Aβ42/Aβ40(+) or Aβ42/Aβ40(−) by applying a cutoff of 0.241 corresponding to the maximum Youden index.

**Fig. 4:**
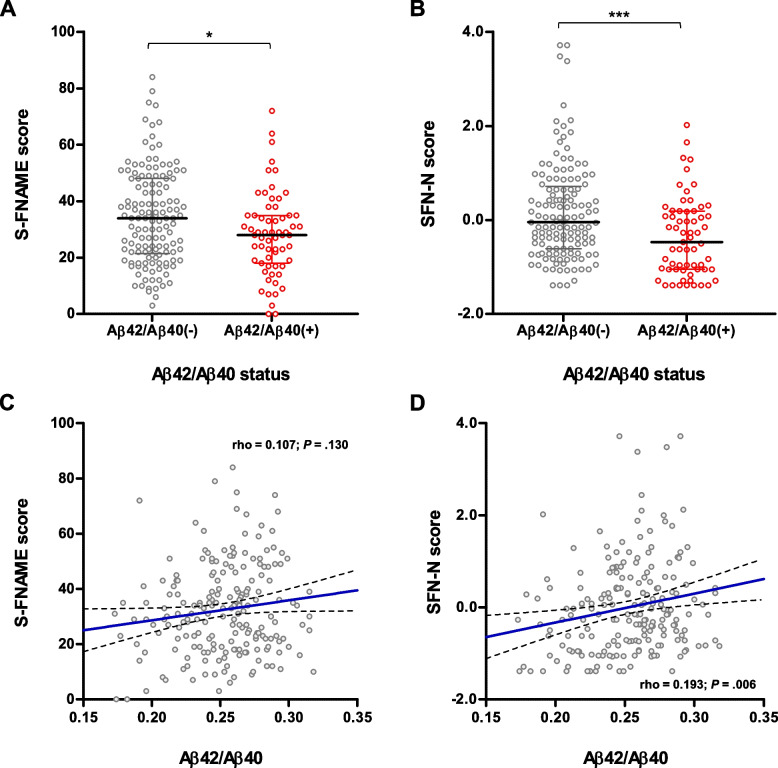
Association of plasma Aβ42/Aβ40 with episodic memory performance. Participants were classified as plasma Aβ42/Aβ40(+) or Aβ42/Aβ40(−) by applying a cutoff of 0.241 corresponding to the maximum Youden index. **A**, **B** Distribution of S-FNAME total score (**A**) and SFN-N composite score (**B**) between Aβ42/Aβ40(−) and Aβ42/Aβ40(+) groups. Cognitive scores were compared between Aβ42/Aβ40(−) and Aβ42/Aβ40(+) groups using Mann-Whitney test. * *P* < .05; *** *P* < .001. Horizontal line depicts median and whiskers depict interquartile range. **C**, **D** Correlations between Aβ42/Aβ40 and S-FNAME total score (**C**) and SFN-N composite (**D**) score. Solid blue line represents the regression line; dashed lines represent 95% confidence interval

Subjects classified as plasma Aβ42/Aβ40(+) performed significantly worse on S-FNAME total score and SFN-N composite score, than those who were Aβ42/Aβ40(−) (*P* = .023 and *P* < .001, respectively) (Fig. 4A and B). A significant positive correlation was found between plasma Aβ42/Aβ40 ratios and the SFN-N composite score (rho = 0.193, *P* < .006), while correlation with S-FNAME total score resulted non-significant (Fig. 4D and C).

Plasma Aβ42/Aβ40 was also associated with brain atrophy, as evidenced by increased ventricular volume in Aβ42/Aβ40(+) individuals in comparison to Aβ42/Aβ40(−) subjects (*P* = .022) (Supplementary Fig. 3A). A trend for reduced hippocampal volume in Aβ42/Aβ40(+) participants was found, although the difference did not reach statistical significance (*P* = .097) (Supplementary Fig. 3B). Correlations between plasma Aβ42/Aβ40 and brain atrophy measures resulted non-significant (Supplementary Fig. 3C–D).

### Association of plasma Aβ42/Aβ40 with longitudinal measures of cognition, brain amyloid deposition and brain atrophy

Participants were followed-up on an annual basis and longitudinal measures of clinical diagnosis, brain amyloid deposition and brain atrophy at V2 were included in this study.

**Fig. 5:**
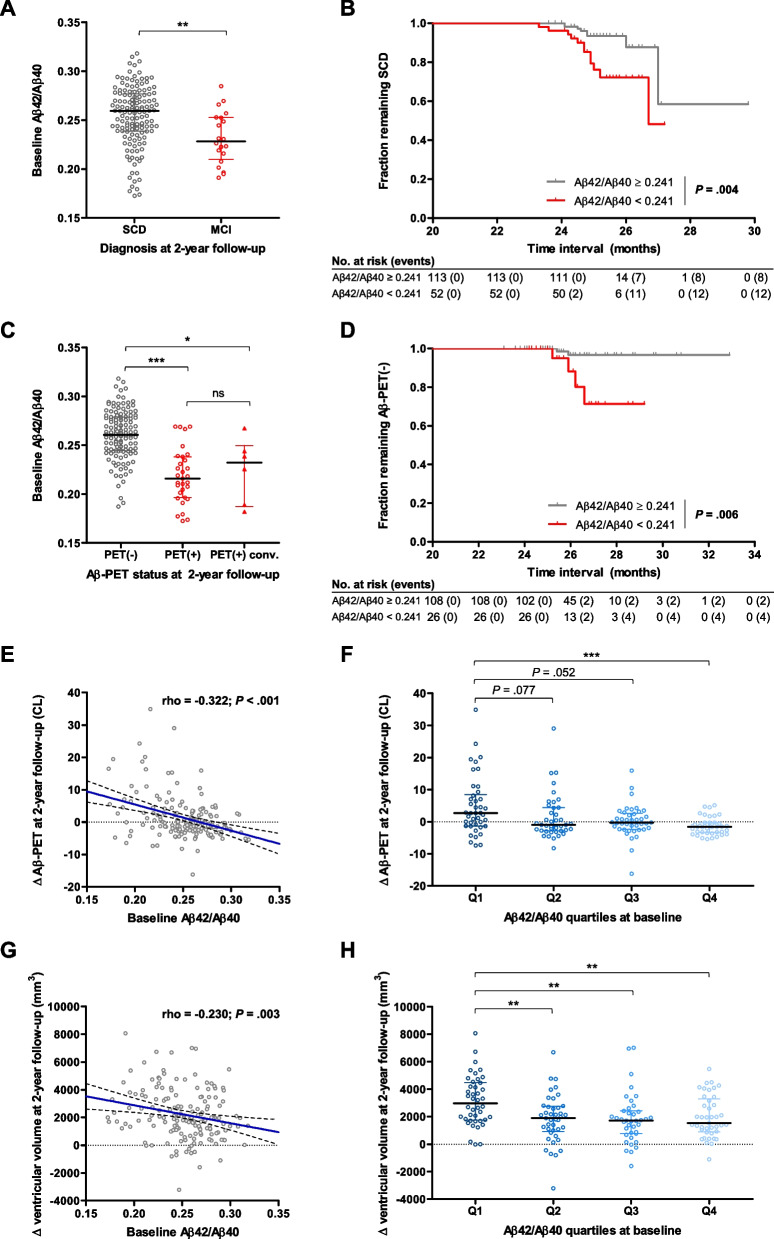
Association of plasma Aβ42/Aβ40 at baseline with longitudinal measures of clinical diagnosis, brain amyloid deposition and brain atrophy at 2-year follow-up. **A** Distribution of plasma Aβ42/Aβ40 levels at baseline between subjective cognitive decline (SCD) and mild cognitive impairment (MCI) individuals at 2-year follow-up. Plasma Aβ42/Aβ40 levels were compared between SCD and MCI groups using Mann Whitney test. ** *P* < .01. Horizontal line depicts median and whiskers depict interquartile range. **B** Kaplan-Meier curves showing fraction of individuals remaining SCD. *P* value of log-rank test is depicted in the lower right. **C** Distribution of plasma Aβ42/Aβ40 levels at baseline between Aβ-PET(−), Aβ-PET(+) and Aβ-PET(+) converter subjects at 2-year follow-up. Aβ-PET(+) converter subjects were defined as individuals who converted from Aβ-PET(−) at baseline to Aβ-PET(+) at 2-year follow-up. Plasma Aβ42/Aβ40 levels were compared among groups using Kruskal-Wallis test followed by the Dunn’s pairwise test with adjustment for multiple comparisons. * *P* < .05; *** *P* < .001. Horizontal line depicts median and whiskers depict interquartile range. **D** Kaplan-Meier curves showing fraction of individuals remaining Aβ-PET(−). *P* value of log-rank test is depicted in the lower right. **E**, **G** Correlations between plasma Aβ42/Aβ40 at baseline and amyloid accumulation (**E**) and brain atrophy (**G**) at 2-year follow-up, as determined by Aβ-PET CL and ventricular volume increments. Solid blue line represents the regression line; dashed lines represent 95% confidence interval. **F**, **H** Distribution of Aβ-PET CL (**F**) and ventricular volume (**H**) increments among the quartiles of plasma Aβ42/Aβ40 at baseline. Aβ-PET CL and ventricular volume increments were compared using Kruskal-Wallis test followed by the Dunn’s pairwise test with adjustment for multiple comparisons. ** *P* < .01; *** *P* < .001. Horizontal line depicts median and whiskers depict interquartile range. Abbreviations: CL, centiloid; Δ, increment; MCI, mild cognitive impairment; ns, non-significant; Q, quartile; SCD, subjective cognitive decline

At this time frame, 20 (12%) of the 165 individuals who remained in the study progressed to MCI. Stratification of participants according to diagnosis at V2 showed significantly lower plasma Aβ42/Aβ40 at baseline in Aβ-PET(+) subjects compared with Aβ-PET(−), both in the SCD and MCI groups (*P* < .001) (Supplementary Fig. 4). Baseline Aβ42/Aβ40 was associated with conversion to MCI, as SCD subjects who converted to MCI at V2 had significantly lower Aβ42/Aβ40 values at baseline than SCD participants who remained stable (*P* = .002) (Fig. 5A). Kaplan-Meier analysis demonstrated that individuals classified as Aβ42/Aβ40(+) at baseline presented an increased risk of progression to MCI than Aβ42/Aβ40(−) subjects (log-rank *P* = .004) (Fig. 5B).

In this subcohort with Aβ-PET data at follow-up, 31 participants were Aβ-PET(+) at baseline, six converted to Aβ-PET(+) over the 2-year period while 128 remained Aβ-PET(−). Noteworthy, Aβ-PET(+) converters presented significantly lower Aβ42/Aβ40 levels at baseline than those who remained Aβ-PET(−) (*P* = .024) (Fig. 5C). Survival curves confirmed an increased risk of conversion to Aβ-PET(+) in Aβ42/Aβ40(+) subjects (log-rank *P* = .006) (Fig. 5D). Consistently, plasma Aβ42/Aβ40 at baseline was significantly correlated with amyloid accumulation at V2 (rho = −0.322, *P* < .001) (Fig. 5E). Individuals with Aβ42/Aβ40 in the lowest quartile at baseline showed greater increases of Aβ-PET CL at 2 years, compared to the other three quartiles (*P* = .077 for Q1 vs Q2, *P* = .052 Q1 vs Q3 and *P* < .001 Q1 vs Q4) (Fig. 5F), suggesting that baseline Aβ42/Aβ40 could be useful for risk-stratification of subjects.

Additionally, plasma Aβ42/Aβ40 at baseline was inversely correlated with progressive brain atrophy as determined by ventricular volume increment (rho = −0.230, *P* = .003) (Fig. 5G). High-risk subjects presenting the lowest Aβ42/Aβ40 ratios at baseline showed greater increases of ventricular volume over 2 years (*P* = .007 for Q1 vs Q2, *P* = .002 Q1 vs Q3 and *P* = .007 Q1 vs Q4) (Fig. 5H). No association was found between baseline plasma Aβ42/Aβ40 and hippocampal volume changes at V2 (data not shown).

## Discussion

In this study, we have presented strong evidence of the clinical performance of a novel MS-based method for the quantification of plasma Aβ42/Aβ40. We have proved that ABtest-MS, which overcomes the drawbacks of other MS-based methods due to its simplified workflow, shows high accuracy, sensitivity and robustness for the detection of early pathological alterations in AD.

As expected and in line with multiple other studies [5, 6, 8, 20, 21], we found decreased plasma Aβ42/Aβ40 values in Aβ-PET positive subjects. This reduction resulted in an 18% difference between groups, compared to the 6–14% variation reported in recent studies comparing multiple plasma Aβ assays [11, 22]. The narrow dynamic range inherent to this plasma biomarker means that the clinical robustness of the Aβ42/Aβ40 ratio could be compromised if very-low-level variability assays are not used in order to assure maximum accuracy [12, 22].

ABtest-MS measures of plasma Aβ42/Aβ40 predicted Aβ-PET status with an AUC of 0.87 and an overall accuracy of 81.5%. These results are similar or slightly superior to those previously reported by IP-MS-based methods in CU subjects, with AUCs ranging from 0.70 to 0.88 [10, 23–27] and accuracies of 72-75% [10, 24, 25]. Recent findings with a novel IP-MS method that removes the digestion step have also shown increased accuracy [28], supporting the hypothesis that simplified workflow results in potentially more robust assays and subsequent better discriminative accuracy.

The cutoff obtained by ABtest-MS at the maximum Youden index to discriminate between Aβ-PET(−) and Aβ-PET(+) subjects is not directly comparable to other cutoff values generated on other technology platforms due to several reasons: (1) ABtest-MS quantify intact full-length Aβ species, whereas other assays quantify truncated plus full Aβ sequence; (2) the extraction method used in ABtest-MS is more aggressive and therefore is less susceptible to the non-covalent interactions of Aβ peptides with other components of the plasma matrix and even antibodies; (3) calibration curves of ABtest-MS are prepared in the same matrix as the samples, i.e. human plasma, in contrast to other assays which use saline solutions; (4) unlike CSF, there are no certified reference materials that would allow different assays to be recalibrated [29].

Concordance analyses between plasma Aβ42/Aβ40 ratio and Aβ-PET status showed a greater number of false positive than false negative determinations, that is, most of the discordant cases (86.5%) were plasma(+)/PET(−). This is in agreement with previous findings reported by our group [18] and others [10, 23, 24], and suggests that Aβ42/Aβ40 values in plasma decline before amyloid brain deposition is detectable by Aβ-PET imaging, and therefore, plasma Aβ42/Aβ40 may be a more sensitive biomarker in perceiving early changes in brain amyloidosis. Accordingly, we also found that subjects who converted to Aβ-PET positivity at 2-year follow-up presented significantly lower baseline plasma Aβ42/Aβ40 ratios than those who remained stable, suggesting that the plasma biomarker may be useful to identify individuals at risk of converting Aβ-PET status in the near future.

By combining the plasma Aβ42/Aβ40 ratio with age, sex and *APOE* ε4 number of alleles, the accuracy for identifying abnormal Aβ-PET status was significantly superior as compared to that of the base model. This means that the predictive ability of the plasma biomarker is additive to the risk information provided by the demographic covariates, and the resulting model achieves an AUC of 0.89. These results confer this biomarker a practical utility as a tool to assist in early diagnosis.

The high sensitivity (86.1%) and negative predictive value (96.4%) obtained suggest that plasma Aβ42/Aβ40 measured with this novel high-sensitivity assay could be a valuable pre-screening tool for identification of potentially eligible subjects for clinical trials or anti-amyloid therapies, such as the recently conditional approved by FDA, Aduhelm [30], or other upcoming treatments. Particularly, the implementation of a pre-screening step would be especially relevant in AD secondary prevention trials, where the low frequency of amyloid pathology in CU individuals results in screening failure rates up to 80% [31]. In this context and in agreement with previous observations from different cohorts of CU individuals [8, 10, 23, 32, 33], a pre-screening step with ABtest-MS in a recruitment scenario targeting the Aβ-positive individuals with SCD (assuming the same prevalence of Aβ positivity as in the FACEHBI cohort) could save 63% of the PET scans required, leading to reduced costs and accelerated clinical trial recruitment.

In the present study, we found that low plasma Aβ42/Aβ40 values were associated with worse episodic memory performance on the S-FNAME, a sensitive face-name associative memory test that has been previously associated to higher Aβ deposition in healthy adults with SCD [19]. Accordingly, the association was stronger with the SFN-N composite, the most sensitive subtest at detecting brain amyloid deposition. To our knowledge, this is the first time that plasma Aβ42/Aβ40 is shown to be cross-sectionally associated to cognitive measures in CU individuals with SCD. Previous studies have reported the absence of associations between plasma amyloid and cognitive functioning, probably due to insufficient sensitivity and robustness of the technologies used to quantify Aβ plasma peptides, i.e. immunoassay-based techniques [34, 35].

Additionally, we observed that individuals with low plasma Aβ42/Aβ40 values presented increased ventricular volume and, at some extent, reduced hippocampal volume, suggesting that the plasma biomarker, as determined by this novel method, can detect the first subtle changes in the AD neurodegeneration process. Similarly to previous studies showing no association of plasma Aβ42/Aβ40 with total brain, grey, and white matter volumes in cognitively normal elderly individuals [36], no significant correlations were found between plasma Aβ42/Aβ40 ratio and ventricular and hippocampal volumes in our study population. These observations could be attributed to the very early stage within the AD continuum of the FACEHBI cohort.

FACEHBI is a longitudinal long-term study with repeated evaluations of cognition and biomarkers. We performed an exploratory assessment of the ability of plasma Aβ42/Aβ40 ratio at baseline to predict disease progression over 2 years. We found that individuals who progressed to MCI presented lower baseline levels of plasma Aβ42/Aβ40 than cognitively stable participants. These findings are highly concordant with previous studies reporting strong associations between low plasma Aβ42/Aβ40 values and increased risk of progression to MCI and/or AD dementia in CU individuals [33, 35, 37, 38]. Additionally and in line with other studies, we also found significant associations of plasma Aβ42/Aβ40 at baseline with longitudinal measures of amyloid brain accumulation [23, 32, 33] and brain atrophy [39, 40]. Altogether, these findings suggest that the measurement of plasma Aβ42/Aβ40 could be helpful in clinical practice to predict short-term disease progression in the preclinical stage of AD.

Over the last few years, other blood-based biomarkers, particularly different phosphorylated forms of tau, have emerged with promising results and are under extensive investigation [41–45]. However, recent evidence suggests that plasma Aβ42/Aβ40 could be the most accurate biomarker in the very early stages of the disease [10, 11, 25, 46], in accordance with previous observations in CSF samples in which Aβ42/Aβ40 was the first altered biomarker in the AD continuum [47, 48]. Further studies should address the diagnostic ability of ABtest-MS measures of plasma Aβ42/Aβ40 in combination with other plasma biomarkers at different stages of AD.

Among the strengths of this study are the characteristics of the FACEHBI cohort, a well-defined and homogeneous population comprised of individuals with cognitive complaints but no objective deficits on a standardized neuropsychological battery. In our opinion, this highly relevant population would especially benefit from appropriate screening and monitoring in clinical practice. Another strength is the use of a highly sensitive and robust MS-based technology, together with an extensively standardized pre-analytical protocol, which assures more consistent and reliable measurements [49]. The robustness of the clinical performance of the assay has been proved since the cross-sectional results at V2 were highly consistent.

## Limitations

Limitations of the present study include the relatively modest population size recruited in a single centre, which may preclude the extrapolation of results to a more heterogeneous population-based sample. However, since all cognitive and biomarker assessments have been collected and processed homogeneously, data uniformity is guaranteed. Furthermore, the generalizability of the results has already been demonstrated at some extent in this study since the model established in the FACEHBI cohort was applied to the independent validation DPUK-Korean cohort with equivalent accuracy. Another limitation to be mentioned is that, at this time, only data at 2-year follow-up is available, which is relatively short for expecting progression in subjects with SCD. However, ABtest-MS measures of plasma Aβ42/Aβ40 have shown to be able to capture changes, not only in amyloid deposition, but also in cognition and neurodegeneration within the 2-year time frame. Furthermore, and since the follow-up of the FACEHBI cohort is currently ongoing, valuable longitudinal information about the ability of plasma Aβ42/Aβ40 ratio to predict clinical and neuropathological changes in the AD continuum will be obtained from successive visits.

## Conclusions

In conclusion, in the current study, we have demonstrated that plasma Aβ42/Aβ40, as determined by this innovative MS-based assay, has potential value as an accurate and cost-effective tool to identify individuals in the earliest stages of the AD continuum, supporting its implementation in clinical trials, preventative strategies and clinical practice. Further investigations are needed to validate the longitudinal performance of ABtest-MS.

## Supplementary Information

**Additional file 1: Supplementary Methods. Supplementary Table 1.** Plasma Aβ42/Aβ40 mass spectrometry methods. **Supplementary Table 2.** Sensitivity and parallelism of ABtest-MS. **Supplementary Table 3.** Accuracy and precision of ABtest-MS for ^15^N-Aβ40 and ^15^N-Aβ42 (Calibration curves). **Supplementary Table 4.** Accuracy and precision of ABtest-MS for ^15^N-Aβ40 and ^15^N-Aβ42 (Quality Control samples). **Supplementary Table 5.** Participant characteristics of the FACEHBI cohort at two-year follow-up. **Supplementary Table 6.** Participant characteristics of the validation cohort (DPUK-Korea). **Supplementary Figure 1.** ROC curves of plasma Aβ40 and Aβ42 for identifying Aβ-PET status. **Supplementary Figure 2.** Diagnostic performance of plasma Aβ42/Aβ40 at two-year follow-up. **Supplementary Figure 3.** Association of plasma Aβ42/Aβ40 with brain atrophy. **Supplementary Figure 4.** Association of plasma Aβ42/Aβ40 at baseline with clinical diagnosis and Aβ-PET status at two-year follow-up. **Supplementary References.**

## Data Availability

The datasets generated and/or analysed during the current study can be made available by the corresponding author upon approved reasonable request.

## Abbreviations

Aβ: Amyloid-β
AD: Alzheimer’s disease
APOE: Apolipoprotein E
AUC: Area under the ROC curve
CI: Confidence interval
CL: Centiloid
FBB: ^18^F-Florbetaben
IP-MS: Immunoprecipitation-MS method
MS: Mass spectrometry
MCI: Mild cognitive impairment
MRI: Magnetic resonance imaging
PET: Positron emission tomography
ROC: Receiver operating characteristic
SCD: Subjective cognitive decline
S-FNAME: Spanish version of the Face-Name Associative Memory Exam
SFN-N: Derived composite S-FNAME Name
