# Supplementary material for: Clinical performance of an antibody-free assay for plasma Aβ42/Aβ40 to detect early alterations of Alzheimer’s disease in individuals with subjective cognitive decline
Source: Alzheimers Res Ther. 2023 Jan 5;15:2. doi: 10.1186/s13195-022-01143-z (PMC9814201; doi:10.1186/s13195-022-01143-z)
Supplement: Supplementary file 1 — Additional file 1: Supplementary Methods. Supplementary Table 1. Plasma Aβ42/Aβ40 mass spectrometry methods. Supplementary Table 2. Sensitivity and parallelism of ABtest-MS. Supplementary Table 3. Accuracy and precision of ABtest-MS for 15N-Aβ40 and 15N-Aβ42 (Calibration curves). Supplementary Table 4. Accuracy and precision of ABtest-MS for 15N-Aβ40 and 15N-Aβ42 (Quality Control samples). Supplementary Table 5. Participant characteristics of the FACEHBI cohort at two-year follow-up. Supplementary Table 6. Participant characteristics of the validation cohort (DPUK-Korea). Supplementary Figure 1. ROC curves of plasma Aβ40 and Aβ42 for identifying Aβ-PET status. Supplementary Figure 2. Diagnostic performance of plasma Aβ42/Aβ40 at two-year follow-up. Supplementary Figure 3. Association of plasma Aβ42/Aβ40 with brain atrophy. Supplementary Figure 4. Association of plasma Aβ42/Aβ40 at baseline with clinical diagnosis and Aβ-PET status at two-year follow-up. Supplementary References. [file 13195_2022_1143_MOESM1_ESM.docx]

**Supplementary Information**

**Supplementary Methods**

***APOE* genotyping**

Genomic DNA was extracted from peripheral blood using the commercially available Chemagic system (Perkin Elmer). The *APOE* genotypes were determined using the Axiom SP array (Thermo Fisher) [1,2]. Ace Alzheimer Center Barcelona has this variable as a standard one in its assessment protocols. Alternatively, the *APOE* genotypes were determined using fluorogenic allele-specific oligonucleotide probes (TaqMan assay; Life Technologies, Spain) for rs7412 (Test ID: C____904973_10) and rs429358 (Test ID: C___3084793_20). For the TaqMan assays, PCR and real-time fluorescence measurements were carried out in QuantStudio3 real-time PCR system (Thermo Fisher Scientific, Spain) using the TaqMan Universal Master Mix (Life Technologies, Spain) methodology according to manufacturer’s instructions. The polymerase chain reaction was performed as follows: first, a pre-read step for 30 s at 60°C, a denaturation for 10 min at 95°C, followed by 40 cycles at 95°C for 15 s and 60°C for 1 min, and a post read stage for 30 s at 60ºC. The genotype was determined using the Genotyping App for Thermo Fisher Cloud by clustering analysis.

**Neuropsychological assessment**

As detailed elsewhere [3], subjects from the FACEHBI cohort were administered an extensive neuropsychological assessment which included the Neuropsychological battery of Fundació ACE (NBACE) [4,5] and additional tests such as the Spanish version of the Face-Name Associative Memory Exam (S-FNAME) [6]. The FNAME is an associative memory test created to detect memory deficits in individuals with preclinical AD [7].

As detailed elsewhere [6], the test begins with an exposure to 16 faces. Participants were shown four faces to a page, one face in each quadrant. They were asked to look at each face for a total of two seconds until they had seen all 16 faces. To control the time, the examiner used his/her finger to point to each one of the 16 faces for two seconds. The participant had to read the name below and try to learn each face–name pair. The same procedure was repeated with the 16 face–occupation pairs. In the Initial study of face–name pairs (FN–N), individuals were then presented the same 16 faces with names underneath and were asked to study the name that goes with the face. Participants were given only one exposure to learn all 16 FN–N pairs. In the Initial cued recall of face–name pairs, they were then shown the face and were asked to recall the name that goes with the face. The correct number of FN–N pairs was recorded as an initial learning score for names (ILN). In the Initial study of face–occupation pairs (FN–O), participants were shown the same faces, but this time with occupations underneath. The FN–O pairs were presented in the same manner as the FN–N pairs until all 16 FN–O pairs were studied. In the Initial cued recall of face–occupation pairs, participants were again shown the face and were asked to recall the occupation that goes with the face. Correct recall of FN–O pairs was tabulated as initial learning of occupations (ILO). In the Immediate cued recall, individuals were shown the face and were asked to recall the name (CRN) and occupation (CRO) that was associated with the face. In the 30-minute delayed cued recall, participants were again presented the face and were asked to recall the name (CRN30) and occupation (CRO30) associated with the face. Considering that long-term memory (CRN30 and CRO30) had to be assessed 30 min after the initial subtests, the S-FNAME lasted for 35-40 min.

Score for each S-FNAME subscale (ILN, ILO, CRN, CRO, CRN30, and CRO30) ranged from 0 to 16, subtotal scores for names (FN–N + ILN + CRN + CRN30) and occupations (FN–O + ILO + CRO + CRO30) were out of 48, and total score for S-FNAME (ILN + ILO + CRN + CRO + CRN30 + CRO30) was out of 96. As detailed previously [8], two S-FNAME composite scores (SNF-N and SNF-O) were obtained after introducing all variables of the S-FNAME and the Word List Learning test from the Wechsler Memory Scale-III in a Principal Component Analysis.

For the aim of the present study, data from S-FNAME total score and the derived composite SFN-N [8] were used for analyses.

**Clinical diagnosis**

At each longitudinal visit of the FACEHBI study, a clinical diagnosis was assigned to each participant according to the information gathered by the neurologist and performance on NBACE [4,5]. Of note, S-FNAME scores were not used for clinical diagnosis. Diagnosis of MCI was defined using Petersen’s [9] and the Cardiovascular health and cognition study [10] criteria.

**Supplementary Table 1 Plasma Aβ42/Aβ40 mass spectrometry methods**

|  | **Araclon Biotech** | **Washington University ^[11,12]^** | **Shimadzu ^[13]^** | **University of Gothenburg ^[14]^** |
| --- | --- | --- | --- | --- |
| **Method** | LC-MS/MS | IP-LC-MS/MS | IP-MALDI/TOF | IP-LC-MS/MS |
| **Sample preparation** | Direct extraction | IP and digestion | Double IP | IP |
| **Antibodies** | NA | HJ5.1 | 6E10 | 4G8 and 6E10 |
| **Sample volume** | 0.2 mL | 0.45 mL | 0.25 mL | 0.25 mL |
| **HPLC** | M3 Micro LC system (Sciex) | Acquity UPLC M-Class (Waters) | NA | UltiMate™ 3000 system (Thermo Fisher Scientific) |
| **Mass spectrometer** | QTRAP 6500+ fitted with SelexION^+^ ion mobility interface (Sciex) | Orbitrap Fusion Lumos (Thermo Fisher Scientific) | AXIMA Performance (Shimadzu/  KRATOS) | Q Exactive^TM^ Quadrupole-Orbitrap^TM^ (Thermo Fisher Scientific) |
| **Calibration curves** |  |  |  |  |
| **Range (pg/ml)** | Aβ40: 50–1000  Aβ42:10–200 | Aβ40: 24.3–1558  Aβ42: 3.6–235 | Aβ40: 43–693  Aβ42: 11–180 | Aβ40: 20–400  Aβ42: 5–100 |
| **Matrix** | Human plasma | PBS and BSA | PBS and BSA | PBS and BSA |

Abbreviations: BSA, bovine serum albumin; HPLC: high-performance liquid chromatography; IP; immunoprecipitation; NA: not applicable; PBS, phosphate buffered saline.

**Supplementary Table 2 Sensitivity and parallelism of ABtest-MS**

|  | **ABtest-MS**  **^15^N-Aβ40** | **ABtest-MS**  **^15^N-Aβ42** |
| --- | --- | --- |
| **Sensitivity** |  |  |
| *LOQ* | 50 pg/ml | 10 pg/ml |
| *Intra-assay RE* | -0.4 to 5.3% | -2.0 to -1.0% |
| *Inter-assay RE* | 0.3% | -1.0% |
| *Intra-assay CV* | 4.1 to 9.0% | 9.9 to 13.2% |
| *Inter-assay CV* | 7.0% | 11.0% |
| **Parallelism** |  |  |
| *Bias* | -6.5 to -4.0% | -5.5 to -2.7% |

Abbreviations: CV, coefficient of variation; LOQ, limit of quantification; RE, relative error.

**Supplementary Table 3 Accuracy and precision of ABtest-MS for ^15^N-Aβ40 and ^15^N-Aβ42 (Calibration curves)**

**A**

|  | **Concentration of ^15^N-Aβ40 (pg/ml)** | | | | | | |  |
| --- | --- | --- | --- | --- | --- | --- | --- | --- |
|  | **50** | **100** | **250** | **400** | **500** | **750** | **1000** | **r** |
| **Run 001** | 51.4 | 100.5 | 250.5 | 404.1 | 521.8 | 769.9 | 994.5 | 0.99961 |
|  | 49.2 | 95.3 | 250.9 | 390.7 | 507.4 | 734.0 | 980.0 |  |
| **Run 002** | 47.8 | 107.6 | 269.5 | 408.1 | 523.2 | 787.7 | 1001.8 | 0.99881 |
|  | 46.0 | 99.5 | 242.8 | 393.6 | 490.5 | 716.9 | 965.1 |  |
| **Run 003** | 48.6 | 106.3 | 256.6 | 404.1 | 503.5 | 762.9 | 1004.5 | 0.99895 |
|  | 51.4 | 100.1 | 237.7 | 381.3 | 488.4 | 705.5 | 1049.2 |  |
| **Mean** | **49.1** | **101.5** | **251.3** | **397.0** | **505.8** | **746.1** | **999.2** | **0.99912** |
| **SD** | **2.1** | **4.6** | **11.1** | **10.2** | **14.8** | **32.3** | **28.6** | - |
| **%CV** | **4.3** | **4.5** | **4.4** | **2.6** | **2.9** | **4.3** | **2.9** | - |
| **%RE** | **-1.9** | **1.5** | **0.5** | **-0.8** | **1.2** | **-0.5** | **-0.1** | - |

**B**

|  | **Concentration of ^15^N-Aβ42 (pg/ml)** | | | | | | |  |
| --- | --- | --- | --- | --- | --- | --- | --- | --- |
|  | **10** | **20** | **50** | **75** | **100** | **150** | **200** | **r** |
| **Run 001** | 11.1 | 21.2 | 46.5 | 81.1 | 100.1 | 151.1 | 206.3 | 0.99890 |
|  | 9.7 | 18.5 | 49.4 | 70.8 | 97.0 | 148.0 | 199.3 |  |
| **Run 002** | 8.6 | 21.5 | 53.5 | 75.4 | 105.7 | 155.1 | 197.1 | 0.99845 |
|  | 9.8 | 21.6 | 45.3 | 73.3 | 100.1 | 152.9 | 190.1 |  |
| **Run 003** | 10.9 | 20.6 | 52.5 | 75.6 | 103.1 | 146.7 | 204.4 | 0.99930 |
|  | 9.7 | 18.7 | 46.7 | 75.0 | 96.3 | 147.2 | 202.7 |  |
| **Mean** | **10.0** | **20.3** | **49.0** | **75.2** | **100.4** | **150.2** | **200.0** | **0.99888** |
| **SD** | **0.9** | **1.4** | **3.4** | **3.4** | **3.6** | **3.4** | **5.9** | - |
| **%CV** | **9.2** | **7.0** | **6.9** | **4.5** | **3.6** | **2.3** | **2.9** | - |
| **%RE** | **-0.4** | **1.7** | **-2.0** | **0.2** | **0.4** | **0.1** | **0.0** | - |

Abbreviations: CV, coefficient of variation; RE, relative error; SD, standard deviation.

Back-calculated concentrations for ^15^N-Aβ40 (A) and ^15^N-Aβ42 (B) at seven concentration levels in the calibration curves, along three analytical runs, are shown. Two calibration curves were analyzed per duplicate, at the beginning and at the end of each analytical sequence. Correlation coefficients correspond to each duplicated calibration curve. The inverse of the theoretical concentration (1/x) was chosen as weighting factor.

**Supplementary Table 4 Accuracy and precision of ABtest-MS for ^15^N-Aβ40 and ^15^N-Aβ42 (Quality Control samples)**

**A**

|  | **Concentration of ^15^N-Aβ40 (pg/ml)** | | |
| --- | --- | --- | --- |
|  | **150** | **400** | **750** |
| **Run 001** | 159.1 | 398.1 | 751.4 |
|  | 149.6 | 414.8 | 743.6 |
| **Run 002** | 155.9 | 430.3 | 806.9 |
|  | 161.6 | 429.7 | 754.8 |
| **Run 003** | 168.5 | 426.4 | 837.8 |
|  | 158.2 | 401.3 | 752.4 |
| **Mean** | 158.8 | 416.8 | 774.5 |
| **SD** | 6.2 | 14.4 | 38.5 |
| **%CV** | 3.9 | 3.5 | 5.0 |
| **%RE** | 5.9 | 4.2 | 3.3 |

**B**

|  | **Concentration of ^15^N-Aβ42 (pg/ml)** | | |
| --- | --- | --- | --- |
|  | **30** | **75** | **150** |
| **Run 001** | 28.0 | 71.9 | 152.6 |
|  | 33.3 | 66.0 | 137.5 |
| **Run 002** | 28.6 | 73.2 | 150.5 |
|  | 30.1 | 74.9 | 135.5 |
| **Run 003** | 33.8 | 76.6 | 158.7 |
|  | 32.5 | 70.1 | 149.9 |
| **Mean** | 31.1 | 72.1 | 147.5 |
| **SD** | 2.5 | 3.8 | 9.0 |
| **%CV** | 8.1 | 5.2 | 6.1 |
| **%RE** | 3.5 | -3.8 | -1.7 |

Abbreviations: CV, coefficient of variation; RE, relative error; SD, standard deviation.

Back-calculated concentrations for ^15^N-Aβ40 (A) and ^15^N-Aβ42 (B) in Quality Control samples, along three analytical runs, are shown. Three concentration levels were assayed: low (3x Lower Limit of Quantitation [LLOQ]), mid and high, for both analytes. Six Quality Control samples, two per concentration level, were included in each analytical run.

**Supplementary Table 5 Participant characteristics of the FACEHBI cohort at 2-year follow-up^a^**

| **Characteristic** | **Aβ-PET(-)**^b^ | **Aβ-PET(+)**^b^ | ***P* value** |
| --- | --- | --- | --- |
| **Participants,** No. (%) | 128 (78) | 37 (22) |  |
| **Age**, years | 66.0 (61.5-71.0) | 72.0 (69.0-75.0) | **< .0001** |
| **Female,** No. (%) | 84 (66) | 17 (46) | **.0486** |
| ***APOE* ɛ4,** No. (%) |  |  |  |
| 0 alleles | 104 (81) | 19 (51) | **.0008** |
| 1 alleles | 21 (16) | 17 (46) |  |
| 2 alleles | 3 (2) | 1 (3) |  |
| **Duration of education**, years | 16.0 (12.0-19.0) | 16.0 (10.0-18.0) | .5222 |
| **FBB-PET**, CL | -5.0 (-9.7-1.7) | 39.4 (20.7-65.0) | **< .0001** |
| **Plasma Aβ40**^c^, pg/ml | 264.7 (237.6-294.8) | 269.3 (249.7-300.8) | .189 |
| **Plasma Aβ42**^c^, pg/ml | 73.3 (64.8-81.8) | 60.3 (56.4-67.9) | **< .0001** |
| **Plasma Aβ42/Aβ40,** ratio | 0.270 (0.255-0.298) | 0.231 (0.201-0.238) | **< .0001** |

Abbreviations: *APOE*, apolipoprotein E; CL, centiloid; FBB-PET, ^18^F-Florbetaben-PET.

^a^ Data are median values (interquartile range), except for the variables Participants, Female and *APOE* ɛ4 number of alleles which are number of cases (%). Differences between Aβ-PET(-) and Aβ-PET(+) groups were tested using Mann-Whitney and Chi-square tests, as appropriate.

^b^ Aβ-PET status was defined using the cutoff established at 13.5 CL corresponding to early amyloid deposition [15].

^c^ Full-length intact Aβ1-40 and Aβ1-42 were quantified by ABtest-MS.

**Supplementary Table 6 Participant characteristics of the validation cohort (DPUK-Korea)^a^**

| **Characteristic** | **Aβ-PET(-)**^b^ | **Aβ-PET(+)**^b^ | ***P* value** |
| --- | --- | --- | --- |
| **Participants,** No. (%) | 131 (89) | 17 (11) |  |
| **Age**, years | 69.0 (64.5-75.0) | 74.0 (72.0-79.0) | **.0036** |
| **Female,** No. (%) | 88 (67) | 10 (59) | .6800 |
| ***APOE* ɛ4**, No. (%) |  |  |  |
| 0 alleles | 102 (78) | 7 (41) | **.0030** |
| 1 alleles | 26 (20) | 8 (47) |  |
| 2 alleles | 3 (2) | 2 (12) |  |
| **Duration of education**, years | 12.0 (6.0-16.0) | 12.0 (6.0-12.0) | .4210 |
| **Aβ-PET**^c^, dcCL | 2.8 (-2.3-7.3) | 58.6 (41.8-76.0) | **< .0001** |
| **MMSE,** score | 28 (27-30) | 28 (27-29) | .2329 |
| **Plasma Aβ40**^d^, pg/ml | 209.7 (182.7-232.8) | 228.9 (208.2-245.1) | .0662 |
| **Plasma Aβ42**^d^, pg/ml | 60.4 (51.3-67.8) | 51.7 (44.0-60.0) | **.0225** |
| **Plasma Aβ42/Aβ40,** ratio | 0.287 (0.259-0.312) | 0.238 (0.200-0.260) | **< .0001** |

Abbreviations: *APOE*, apolipoprotein E; dcCL, direct comparison centiloid units; MMSE, Mini-Mental State Examination.

^a^ Data are median values (interquartile range), except for the variables Participants, Female and *APOE* ɛ4 number of alleles which are number of cases (%). Differences between Aβ-PET(-) and Aβ-PET(+) groups were tested using Mann-Whitney and Chi-square tests, as appropriate.

^b^ Aβ-PET status was defined as previously described [16].

^c^ Participants underwent either ^18^F-Florbetaben (FBB) (N=23) or ^18^F-Flutemetamol (FMM) PET (N=125).

^d^ Full-length intact Aβ1-40 and Aβ1-42 were quantified by ABtest-MS.

**Supplementary Figure 1 ROC curves of plasma Aβ40 and Aβ42 for identifying Aβ-PET status**

**
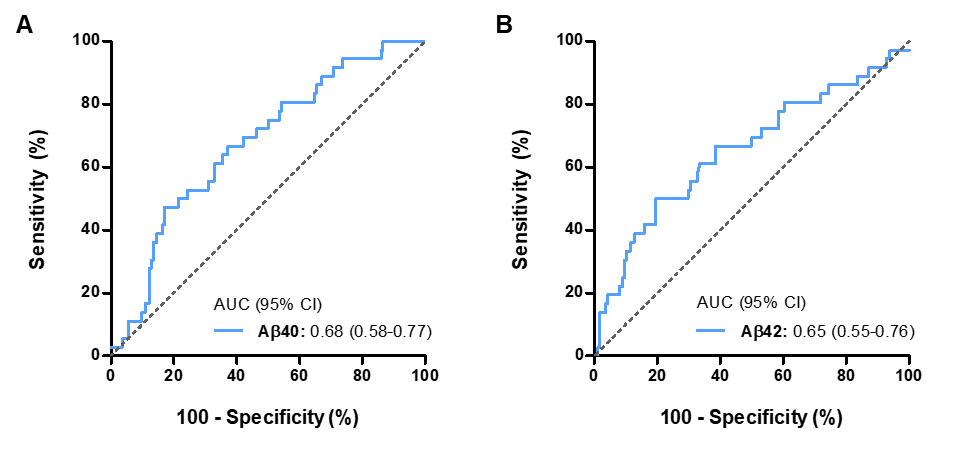
**

**(A-B**) Receiver operating characteristic (ROC) curves for discriminating Aβ-PET status. ROC curves are shown for plasma Aβ40 (A) and Aβ42 (B).

Abbreviations: AUC, area under the curve; CI, confidence interval.

**Supplementary Figure 2 Diagnostic performance of plasma Aβ42/Aβ40 at 2-year follow-up**


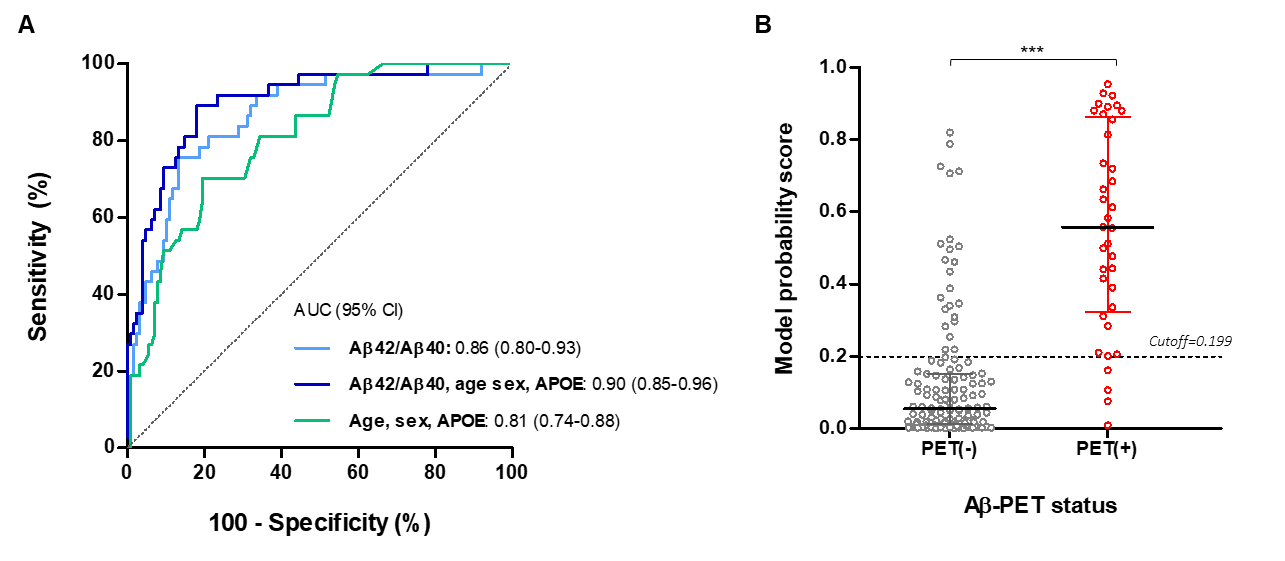


**(A)** Receiver operating characteristic (ROC) curves for discriminating Aβ-PET status at 2-year follow-up. ROC curves are shown for plasma Aβ42/Aβ40, plasma Aβ42/Aβ40 adjusted with age, sex and *APOE* ɛ4 number of alleles, and the demographic model including only age, sex and *APOE* ɛ4 number of alleles.

**(B)** Probability scores distribution derived from the full logistic regression model (Aβ42/Aβ40, age, sex *APOE* ɛ4 number of alleles) to predict Aβ-PET status, between Aβ-PET(-) and Aβ-PET(+) groups. Model probability scores were compared between Aβ-PET(-) and Aβ-PET(+) groups using Mann-Whitney test. *** *P* < .001. Horizontal line depicts median and whiskers depict interquartile range.

Abbreviations: *APOE*: apolipoprotein E; AUC, area under the curve; CI, confidence interval.

**Supplementary Figure 3 Association of plasma Aβ42/Aβ40 with brain atrophy**


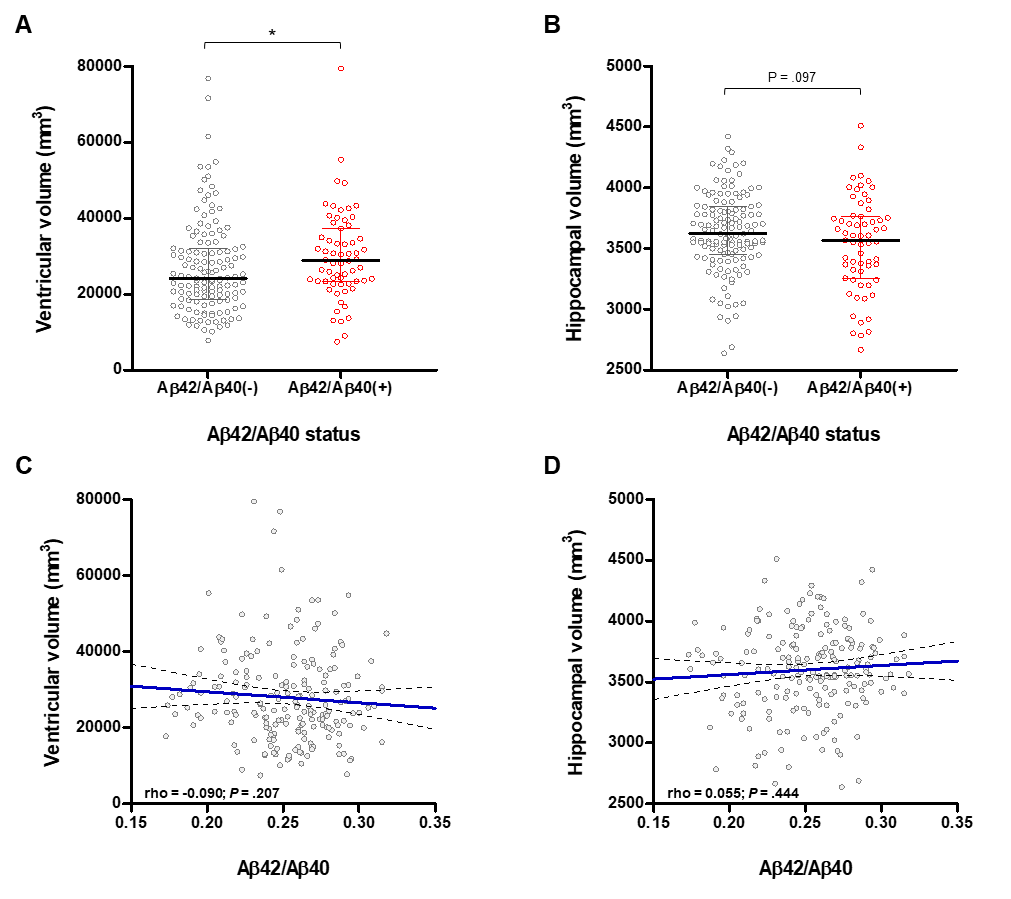


Participants were classified as plasma Aβ42/Aβ40(+) or Aβ42/Aβ40(-) by applying a cutoff of 0.241 corresponding to the maximum Youden index. Volume data correspond to regional volume corrected by total intracranial volume.

**(A-B)** Distribution of normalized ventricular (A) and hippocampal (B) volume between Aβ42/Aβ40(-) and Aβ42/Aβ40(+) groups. Normalized regional volumes were compared between Aβ42/Aβ40(-) and Aβ42/Aβ40(+) groups using Mann-Whitney test. * *P* < .05. Horizontal line depicts median and whiskers depict interquartile range.

**(C-D)** Correlations between Aβ42/Aβ40 and normalized ventricular (C) and hippocampal (D) volume. Solid blue line represents the regression line; dashed lines represent 95% confidence interval.

**Supplementary Figure 4 Association of plasma Aβ42/Aβ40 at baseline with clinical diagnosis and Aβ-PET status at 2-year follow-up**

**
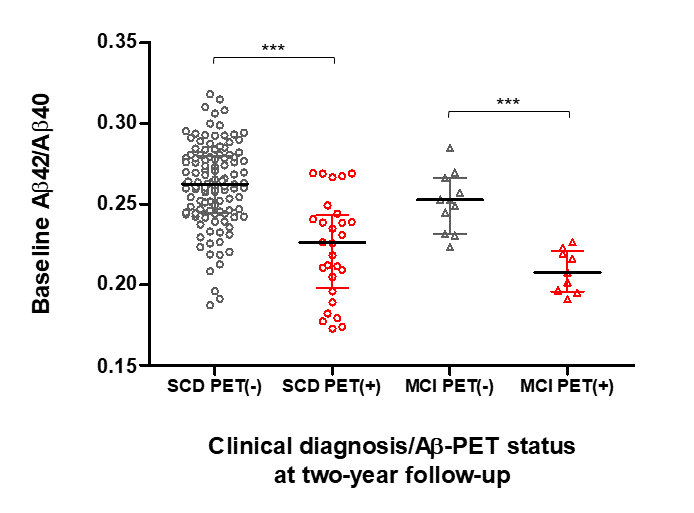
**

Distribution of plasma Aβ42/Aβ40 levels at baseline according to the clinical diagnosis and Aβ-PET status at 2-year follow-up. Plasma Aβ42/Aβ40 levels were compared between SCD PET(-) and SCD PET(+) groups, and between MCI PET(-) and MCI PET(+) groups using Mann-Whitney test. *** *P* < .001. Horizontal line depicts median and whiskers depict interquartile range.

Abbreviations: MCI, mild cognitive impairment; SCD, subjective cognitive decline.

**Supplementary References**

SUPPLEMENTARY REFERENCES

1. Moreno-Grau S, de Rojas I, Hernández I, Quintela I, Montrreal L, Alegret M, et al. Genome-wide association analysis of dementia and its clinical endophenotypes reveal novel loci associated with Alzheimer's disease and three causality networks: The GR@ACE project. Alzheimers Dement. 2019;15(10):1333-47.

2. de Rojas I, Moreno-Grau S, Tesi N, Grenier-Boley B, Andrade V, Jansen IE, et al. Common variants in Alzheimer's disease and risk stratification by polygenic risk scores. Nature communications. 2021;12(1):3417.

3. Rodriguez-Gomez O, Sanabria A, Perez-Cordon A, Sanchez-Ruiz D, Abdelnour C, Valero S, et al. FACEHBI: A Prospective Study of Risk Factors, Biomarkers and Cognition in a Cohort of Individuals with Subjective Cognitive Decline. Study Rationale and Research Protocols. The journal of prevention of Alzheimer's disease. 2017;4(2):100-08.

4. Alegret M, Espinosa A, Vinyes-Junqué G, Valero S, Hernández I, Tárraga L, et al. Normative data of a brief neuropsychological battery for Spanish individuals older than 49. Journal of clinical and experimental neuropsychology. 2012;34(2):209-19.

5. Alegret M, Espinosa A, Valero S, Vinyes-Junqué G, Ruiz A, Hernández I, et al. Cut-off scores of a brief neuropsychological battery (NBACE) for Spanish individual adults older than 44 years old. PLoS One. 2013;8(10):e76436.

6. Alegret M, Valero S, Ortega G, Espinosa A, Sanabria A, Hernández I, et al. Validation of the Spanish Version of the Face Name Associative Memory Exam (S-FNAME) in Cognitively Normal Older Individuals. Archives of clinical neuropsychology : the official journal of the National Academy of Neuropsychologists. 2015;30(7):712-20.

7. Rentz DM, Amariglio RE, Becker JA, Frey M, Olson LE, Frishe K, et al. Face-name associative memory performance is related to amyloid burden in normal elderly. Neuropsychologia. 2011;49(9):2776-83.

8. Sanabria A, Alegret M, Rodriguez-Gomez O, Valero S, Sotolongo-Grau O, Monté-Rubio G, et al. The Spanish version of Face-Name Associative Memory Exam (S-FNAME) performance is related to amyloid burden in Subjective Cognitive Decline. Scientific reports. 2018;8(1):3828.

9. Petersen RC. Mild cognitive impairment as a diagnostic entity. Journal of internal medicine. 2004;256(3):183-94.

10. Lopez OL, Jagust WJ, DeKosky ST, Becker JT, Fitzpatrick A, Dulberg C, et al. Prevalence and classification of mild cognitive impairment in the Cardiovascular Health Study Cognition Study: part 1. Arch Neurol. 2003;60(10):1385-9.

11. Ovod V, Ramsey KN, Mawuenyega KG, Bollinger JG, Hicks T, Schneider T, et al. Amyloid β concentrations and stable isotope labeling kinetics of human plasma specific to central nervous system amyloidosis. Alzheimers Dement. 2017;13(8):841-49.

12. Hu Y, Kirmess KM, Meyer MR, Rabinovici GD, Gatsonis C, Siegel BA, et al. Assessment of a Plasma Amyloid Probability Score to Estimate Amyloid Positron Emission Tomography Findings Among Adults With Cognitive Impairment. JAMA network open. 2022;5(4):e228392.

13. Nakamura A, Kaneko N, Villemagne VL, Kato T, Doecke J, Doré V, et al. High performance plasma amyloid-β biomarkers for Alzheimer's disease. Nature. 2018;554(7691):249-54.

14. Keshavan A, Pannee J, Karikari TK, Rodriguez JL, Ashton NJ, Nicholas JM, et al. Population-based blood screening for preclinical Alzheimer's disease in a British birth cohort at age 70. Brain. 2021;144(2):434-49.

15. Bullich S, Roé-Vellvé N, Marquié M, Landau SM, Barthel H, Villemagne VL, et al. Early detection of amyloid load using (18)F-florbetaben PET. Alzheimers Res Ther. 2021;13(1):67.

16. Jang H, Kim JS, Lee HJ, Kim CH, Na DL, Kim HJ, et al. Performance of the plasma Aβ42/Aβ40 ratio, measured with a novel HPLC-MS/MS method, as a biomarker of amyloid PET status in a DPUK-KOREAN cohort. Alzheimers Res Ther. 2021;13(1):179.
